# Which aspects of anhedonia predict response to pharmacotherapy in major depressive disorder?

**DOI:** 10.1017/S0033291725102894

**Published:** 2025-12-29

**Authors:** Rudolf Uher, Sakina J. Rizvi, Lena C. Quilty, Barbara Pavlova, Abraham Nunes, Jane A. Foster, Raymond W. Lam, Roumen Milev, Daniel J. Müller, Valerie Taylor, Claudio N. Soares, Susan Rotzinger, Sidney H. Kennedy, Benicio N. Frey

**Affiliations:** 1 Department of Psychiatry, Dalhousie University, Halifax, NS, Canada; 2 ASR Suicide and Depression Studies Program, Department of Psychiatry, St. Michael’s Hospital, Toronto, Ontario, Canada; 3 Campbell Family Mental Health Research Institute, Centre for Addiction and Mental Health, Toronto, ON, Canada; 4 Department of Psychiatry, University of Toronto, Toronto, ON, Canada.; 5 Faculty of Computer Science, Dalhousie University, Halifax, NS, Canada; 6 Department of Psychiatry & Behavioural Neurosciences, McMaster University, Hamilton, ON, Canada; 7 Center for Depression Research and Clinical Care, Department of Psychiatry, Peter O’Donnell Jr. Brain Institute, UT Southwestern Medical Center, Dallas, TX, USA; 8 Department of Psychiatry, University of British Columbia, Vancouver, British Columbia, Canada; 9 Department of Psychiatry, Providence Care, Queen’s University, Kingston, ON, Canada; 10 Centre for Mental Health Sciences, Ontario Shores, Whitby, ON, Canada; 11 Cumming School of Medicine, Department of Psychiatry, University of Calgary, Calgary, AB, Canada; 12 Department of Psychiatry & Behavioural Neurosciences, McMaster University, Hamilton, ON, Canada; 13 Mood Disorders Program and Women’s Health Concerns Clinic, St. Joseph’s Healthcare Hamilton, ON, Canada; 14 Homewood Research Institute, Guelph, ON, Canada

**Keywords:** anhedonia, antidepressant medication, major depressive disorder, predictors of treatment response

## Abstract

**Background:**

Anhedonia is a multidimensional concept, and it is not known which aspects of it are linked to the heterogeneity of treatment responses in major depressive disorder (MDD). We examine the role of anhedonia dimensions in predicting response to antidepressant medication and adjunctive pharmacotherapy.

**Methods:**

In CAN-BIND-1, 187 adults with MDD completed the Dimensional Anhedonia Rating Scale (DARS) and the Snaith–Hamilton Pleasure Scale (SHAPS) before undergoing 8 weeks of treatment with escitalopram. At week 8, 90 nonresponders received adjunctive treatment with aripiprazole for an additional 8 weeks. Mixed-effects models tested the hobbies, food, social, and sensory subscales and items of DARS and SHAPS as predictors of change in the Montgomery-Åsberg Depression Rating Scale (MADRS).

**Results:**

Of the four DARS subscales, sensory anhedonia predicted a worse treatment outcome with escitalopram (*b* = 1.14, 95%CI 0.08 to 2.20, *p* = 0.034) as did a three-item SHAPS sensory anhedonia subscale (*b* = 1.50, 95%CI 0.43 to 2.57, *p* = 0.006). A combined DARS–SHAPS sensory anhedonia subscale complemented the previously reported interest–activity symptom dimension to improve treatment outcome prediction. In contrast, food and social anhedonia dimensions predicted worse outcomes with adjunctive aripiprazole (*b* = 2.52, 95%CI 1.25 to 3.80, *p* < 0.001; *b* = 2.56, 95%CI 1.16 to 3.96, *p* < 0.001). Corresponding SHAPS items showed similar results.

**Conclusions:**

The inability to enjoy sensory experiences and the lack of interest in food and social activities distinctly predict outcomes with serotonergic versus dopaminergic pharmacotherapy. These findings require replication and extension to other treatments.

## Introduction

Anhedonia is a multifaceted concept that is relevant to the understanding of major depressive disorder (MDD) and individual differences in response to treatments.

Originally described as the inability to experience pleasure (Ribot, 1896), the concept of anhedonia has expanded to include dysfunctions at all stages of the reward process (De Fruyt, Sabbe, & Demyttenaere, 2020). Modern definitions of anhedonia cover deficiencies in interest, motivation, effort, engagement, and enjoyment (Pizzagalli, 2022). The nature and extent of anhedonia vary from person to person in multiple ways, so a complete characterization of its phenomenology requires measurement along multiple dimensions (Wang, Leri, & Rizvi, 2022). Aspects of anhedonia can be classified according to the reward process or the reward domain. Process-based classification involves distinctions between missing enjoyment (consummatory anhedonia, ‘liking’), lacking interest or motivation (anticipatory anhedonia, ‘wanting’), and failure to make associations between behaviors and rewarding outcomes, ‘learning’ (Berridge & Robinson, 2003). Classification by the domain of reward distinguishes between primary rewards (e.g., food), other physical rewards (e.g., pleasant sensory experiences), social rewards (e.g., going out, meeting people for conversation or play), and nonphysical secondary (e.g., monetary) rewards. While mechanistic models emphasize the reward process, psychometric analyses of human data suggest that interpersonal differences in anhedonia cluster primarily by reward domain (Case et al., 2022; Rizvi et al., 2015). Rizvi and colleagues developed a self-report measure of anhedonia that samples all stages of the reward process across multiple domains of potentially rewarding experiences and activities (Rizvi et al., 2015). A psychometric analysis of data obtained with this measure revealed dimensions of leisure activities, food, socializing, and sensory experiences (Rizvi et al., 2015). This four-domain structure has been reproduced across studies using the same measure (Arrua-Duarte et al., 2019; Gorostowicz et al., 2023), another validated measure of anhedonia (Fresán & Berlanga, 2013; Zhang et al., 2021), or multiple measures of anhedonia and related concepts (Case et al., 2022). Although its multidimensional structure is now established, clinical applications have applied anhedonia as a single score without distinguishing reward domains. The present study examines whether dimensions of anhedonia have distinct roles in determining responses to treatments.

Contemporary classifications consider anhedonia to be a cardinal symptom of MDD (APA, 2022; WHO, 2021). Yet, anhedonia is far from a uniform experience among people with depression. One in five adults with MDD does not endorse diminished interest or pleasure as a symptom of their depressive episodes (Mitchell et al., 2009). Others differ in the type, degree, and extent of anhedonia they experience (Pizzagalli, 2022). As a result, anhedonia is a major contributor to the clinical heterogeneity of MDD (Fried, Coomans, & Lorenzo-Luaces, 2020). Profound loss of interest and inability to experience pleasure may be a marker of a type of MDD that is associated with poor outcomes. Specifically, higher levels of anhedonia predict the persistence of depression, lack of functional recovery, and suicide attempts among adolescents and adults with MDD (Fawcett et al., 1990; Gabbay et al., 2015; Luca et al., 2024; Sagud et al., 2021; Spijker, Bijl, de Graaf, & Nolen, 2001; Vinckier, Gourion, & Mouchabac, 2017). Elevated anhedonia scores have been reported to predict less improvement with antidepressant medication (McMakin et al., 2012; Vrieze et al., 2014), cognitive-behavioral therapy (Alsayednasser et al., 2022; Craske et al., 2019), behavioral activation (Alsayednasser et al., 2022), and repetitive transcranial magnetic stimulation (Downar et al., 2014). However, significant discrepancies in the literature suggest the need for systematic examination before anhedonia can be used as an indicator in treatment planning. In one large study, anhedonia predicted a worse response to antidepressant medication and cognitive-behavioral therapy in univariate analyses, but the effects disappeared after controlling for distress (Khazanov et al., 2020). In another study, only one of two measures of anhedonia predicted response to antidepressant medication (Dunlop et al., 2020). The divergence of predictive effects depending on which measure is used or what other symptoms are controlled for suggests that the exact components that determine therapeutic outcomes remain to be delineated. We hypothesize that a specific dimension of anhedonia, rather than anhedonia overall, may predict antidepressant treatment outcomes. We are aware of no published study that examined the four domain-based dimensions of anhedonia as predictors of treatment response. Therefore, we aim to test the leisure activities, food, social, and sensory anhedonia dimensions as predictors of response to antidepressant treatment.

The most direct clinical application of anhedonia may lie in indicating that a specific treatment could be more effective than an alternative for a given person with MDD. Such differential treatment indications can be traced to neurotransmitters involved in reward processes and antidepressant drug targets. Among these neurotransmitters, dopamine stands out as a key player in reward-related processes of incentive motivation, effort-based decision-making, and salience attribution, which are disrupted in anticipatory anhedonia (Berridge, 2007). The role of dopamine in reward and positive affect has been contrasted with the functions of serotonin in modulating negative affect and anxiety (Lane, 2014). Dopamine and serotonin exert opposing effects on reinforcement learning: dopamine stimulates and serotonin dampens the learning process (Cardozo Pinto et al., 2025). This aligns with the notion that anhedonia predicts worse outcomes with serotonergic antidepressants (McMakin et al., 2012; Vrieze et al., 2014), but good response to dopamine-enhancing medications (Cao et al., 2019; Dormegny-Jeanjean et al., 2023). Yet, the picture is far from clear as behavioral measures of anhedonia predicted response to a dopamine-enhancing medication in the opposite direction (Whitton et al., 2020). Recent experiments implicate dopamine and serotonin as complementary players in the reward process, with their respective roles changing with the time scale and social context (Batten et al., 2024; Cohen, 2015; Rogers, 2011) These findings raise the question of whether different aspects of anhedonia may predict responses to serotonergic vs. dopaminergic medications rather than a unitary anhedonia predicting poor response to serotoninergic and good response to dopaminergic medication. Therefore, we aim to test the four dimensions of anhedonia in predicting response to a serotonergic antidepressant and to an adjunctive dopaminergic treatment.

In addition to anhedonia itself, a related construct of interest–activity symptoms has been used to predict depression treatment outcomes. The interest–activity symptom dimension was derived in a factor analysis of items from three depression rating scales (Uher et al., 2008). It groups items probing interest, ability to feel, enjoyment, concentration, ability to make decisions, energy, engagement in work, and activity. This symptom dimension consistently predicted worse outcome of treatment with antidepressants across three clinical trials (Uher et al., 2012, 2020). While the interest–activity symptoms overlap with anhedonia, they differ in several ways. First, they include energy, concentration, ability to make decisions, engagement in work and level of activity in addition to interest and enjoyment. Second, they concern interest and activity in general without distinguishing domains of interest or activity or specifying potentially rewarding outcomes. Nonetheless, these symptoms include the key anhedonia items, interest and enjoyment, and have been grouped into a dimension because they tend to co-occur among people with MDD. In this study, we aim to examine the relative roles of interest–activity and anhedonia dimensions by testing their unique and overlapping contributions to predicting antidepressant treatment outcomes.

In summary, we propose to advance the understanding of the role of anhedonia and related symptoms in depression treatment by addressing two primary questions. First, we test which dimensions of anhedonia are associated with nonresponse to serotonin-reuptake inhibitors among individuals with MDD. Second, we will test which dimensions of anhedonia are associated with response to adjunctive partial dopamine agonists among those who did not respond to a serotonin-reuptake inhibitor. In addition, we will examine which symptoms of anhedonia complement the existing measure of interest and activity in predicting response to antidepressant medication and adjunctive treatment.

## Methods

### Participants and treatment

We examine the predictive value of anhedonia dimensions among participants in the Canadian Biomarker Integration Network in Depression trial (CAN-BIND-1) recruited at six clinical-academic sites in four Canadian cities (Kennedy et al., 2019; Lam et al., 2016). This dataset included adults with MDD who were in a major depressive episode with moderate to severe symptoms at study entry. Diagnoses of MDD and comorbid mental disorders according to the Diagnostic and Statistical Manual, 4th edition (DSM-IV) were established using the MINI International Neuropsychiatric Interview (Sheehan et al., 1998). Exclusion criteria were a lifetime diagnosis of bipolar disorder, psychosis, suicide risk inconsistent with outpatient treatment, pregnancy, breastfeeding, and failure to respond to four or more adequate trials of antidepressants during the current depressive episode (Lam et al., 2016). CAN-BIND-1 has two phases, each lasting 8 weeks. In phase I, all CAN-BIND-1 participants were offered treatment with the selective serotonin-reuptake inhibitor escitalopram (dose 10–20 mg, median 20 mg), one of the most commonly used antidepressant medications (Lam et al., 2024). In phase II, those who had not reached a response, defined as a reduction of depressive symptoms by 50% or more by treatment week 8, were offered add-on treatment with the partial dopamine agonist aripiprazole (dose 2–10 mg, median 2 mg), a first-line adjunctive treatment option (Lam et al., 2024). Aripiprazole was always used in combination with escitalopram. Through the 16 weeks of treatment, participants attended assessments every two weeks. All participants signed informed consent as approved by the Research Ethics Boards of the host institutions. CAN-BIND-1 is registered at ClinicalTrials.gov (clinicaltrials.gov/study/NCT01655706).

### Measures of anhedonia

We measured anhedonia with two self-report scales. The *Dimensional Anhedonia Rating Scale* (DARS) is a 17-item questionnaire that measures the interest, motivation, effort, and pleasure across four domains of potentially rewarding activities and experiences: hobbies/pastimes, food/drinks, social activities, and sensory experiences (Rizvi et al., 2015). Each domain refers to the participant’s own examples of activities or experiences they would typically engage in or find enjoyable. The participants answered each item on a five-point Likert scale with anchors ‘not at all’ (= 0), ‘slightly’ (= 1), ‘moderately’ (= 2), ‘mostly’ (= 3), and ‘very much’ (= 4), indexing the level of interest, motivation, effort, or enjoyment. We calculated the domain scores *hobbies* (4 items, range 0–16), *food* (4 items, range 0–16), *social* (4 items, range 0–16), and *sensory* anhedonia (5 items, range 0–20), and the total DARS anhedonia score (17 items, range 0–68) so that higher values reflect better hedonic functioning and *lower* values index more severe anhedonia. The second measure of anhedonia was the Snaith-Hamilton Pleasure Scale (SHAPS), a broadly used 14-item self-report measure focused on the consummatory stage of the hedonic process involving pleasure and enjoyment (Snaith et al., 1995). SHAPS does not have established subscales, but its items, selected to minimize cultural bias, cover common pastime activities, food, social, and sensory experiences. Participants answer each item on a four-point Likert scale with anchors ‘definitely agree’ (= 1), ‘agree’ (= 2), ‘disagree’ (= 3), and ‘strongly disagree’ (= 4). While the original publication dichotomized responses (Snaith et al., 1995), most subsequent research has used all four response options (Franken, Rassin, & Muris, 2007; Mathew et al., 2025; Savitz et al., 2025; Schmidt et al., 2024). For consistency with the body of published research and to obtain a range of scores comparable to DARS, we used all four response options, resulting in a total SHAPS anhedonia score ranging from 14 to 56, with *higher* values reflecting more severe anhedonia. Participants completed DARS and SHAPS at baseline and again at week 8.

### Primary outcome measure

The Montgomery and Åsberg Depression Rating Scale (MADRS), a ten-item clinician-rated scale is an established measure of depression severity and the primary outcome scale in CAN-BIND-1 (Montgomery & Asberg, 1979). Study psychiatrists administered the scale during a 15–20 minute interview and used both participants’ answers about symptoms over the past 7 days and their observations to determine each item’s score. The total MADRS score has a potential range of 0–60, with higher scores indicating more severe depressive symptoms.

### The interest–activity symptom dimension

The interest–activity symptoms dimension is derived from selected items of MADRS and the Quick Inventory for Depressive Symptomatology, Self-Report (QIDS-SR), a questionnaire completed by CAN-BIND-1 participants at baseline and week 8. Specifically, we constructed the interest–activity symptom dimension as a sum of 3 MADRS items (concentration, lassitude, inability to feel) and 3 QIDS items (concentration, interest, energy) with QIDS item scores doubled to ensure equal weighting of the clinician-assessed and self-report measurements, as previously described (Uher et al., 2012, 2020). The resulting score ranges from 0 to 36, with higher scores indicating a more severe loss of interest and reduction in activity.

### Data analysis

The analysis started by addressing the primary research questions regarding the predictive value of anhedonia dimensions based on the established DARS subscales, before progressing to explore individual symptom items and examine the complementarity between anhedonia dimensions and interest–activity symptoms. In all the analyses, the dependent variable was the MADRS score at 2, 4, 6, and 8 weeks after initiating treatment. The predictors of interest were measures of anhedonia obtained just before initiating each treatment: at baseline for escitalopram and at week 8 for adjunctive aripiprazole. We used linear mixed-effects models for repeated measures to enable optimal use of information from participants with incomplete sets of measurements without making strong assumptions about missing data. We fitted these models using maximum likelihood estimation, which provides valid results under the missing-at-random assumption without imputing missing values. This approach has been recommended as it provides results that are relatively robust to common sources of bias (Lane, 2008; Siddiqui, 2011; Siddiqui, Hung, & O’Neill, 2009). To make the effects of scales, subscales, and items comparable, we standardized all predictors (by subtracting the mean and dividing by the standard deviation) before testing. We reversed the DARS total and subscale scores so that for all predictors, higher values index more severe symptoms. We report all results as a change in MADRS points per one standard deviation of each predictor.

To answer research question 1, we tested the effects of the four DARS anhedonia subscales (hobbies, food, social, and sensory) on weeks 2–8 MADRS scores among all 187 participants who completed one or more post-baseline measurements while receiving treatment with escitalopram. We tested each DARS subscale in a separate model. We entered baseline MADRS, age, sex, and site as fixed covariates. A random effect of the individual modeled the nonindependence of repeated observations from the same participant.

To answer research question 2, we tested the effects of the four week-8 DARS anhedonia subscales on MADRS at weeks 10–16 among the 90 participants who received adjunctive aripiprazole. We did this in four linear mixed-effects models with week-8 MADRS, age, sex, and site as fixed covariates and a random effect of individual.

To explore the nuanced nature of any predictive effects, we repeated the above mixed-effects models for each of the 17 DARS and 14 SHAPS items, and for the total DARS and SHAPS scores.

Finally, we examined the complementarity of anhedonia subscales with the interest–activity symptom dimension in mixed-effect models that included one anhedonia subscale and the baseline or week-8 interest–activity symptom dimension derived from MADRS and QIDS items obtained at the same time.

We report results as the number of MADRS points change per one standard deviation of each predictor, along with the estimate’s 95% confidence interval (95%CI) and a two-sided *p*-value. Given the present study’s part-hypothesis-driven and part-exploratory nature, we consider the statistical significance and multiple testing separately for the primary research questions’ tests and exploratory analyses. For tests of primary research questions, we consider findings with a *p*-value smaller than 0.05 as nominally significant and findings with a *p*-value smaller than 0.0125 (0.05/4 anhedonia subscales) as statistically significant after correction for multiple testing. For exploratory analyses, we derive a false-discovery rate (FDR) q-value from a list of 31 tests of all SHAPS and DARS items following the Benjamini–Hochberg procedure (Benjamini & Hochberg, 1995). We consider findings with a *p*-value smaller than 0.05 as nominally significant and findings with a Benjamini–Hochberg false discovery rate q-value smaller than 0.1 as significant after correction for multiple comparisons.

The sample size was determined by the original CAN-BIND1 study. A post-hoc power calculation showed that the sample of 187 escitalopram-treated participants provided 80% power to detect an effect size of 0.15 (corresponding to 1.39 MADRS points) or greater as nominally significant and an effect size of 0.17 (1.65 MADRS points) as significant after correction for multiple testing. The sample of 95 aripiprazole-treated participants provided 80% power to detect an effect size of 0.20 (1.9 MADRS points) or greater as nominally significant and an effect size of 0.24 (2.3 MADRS points) as significant after correction for multiple testing.

## Results

### Participation

**Figure 1.**
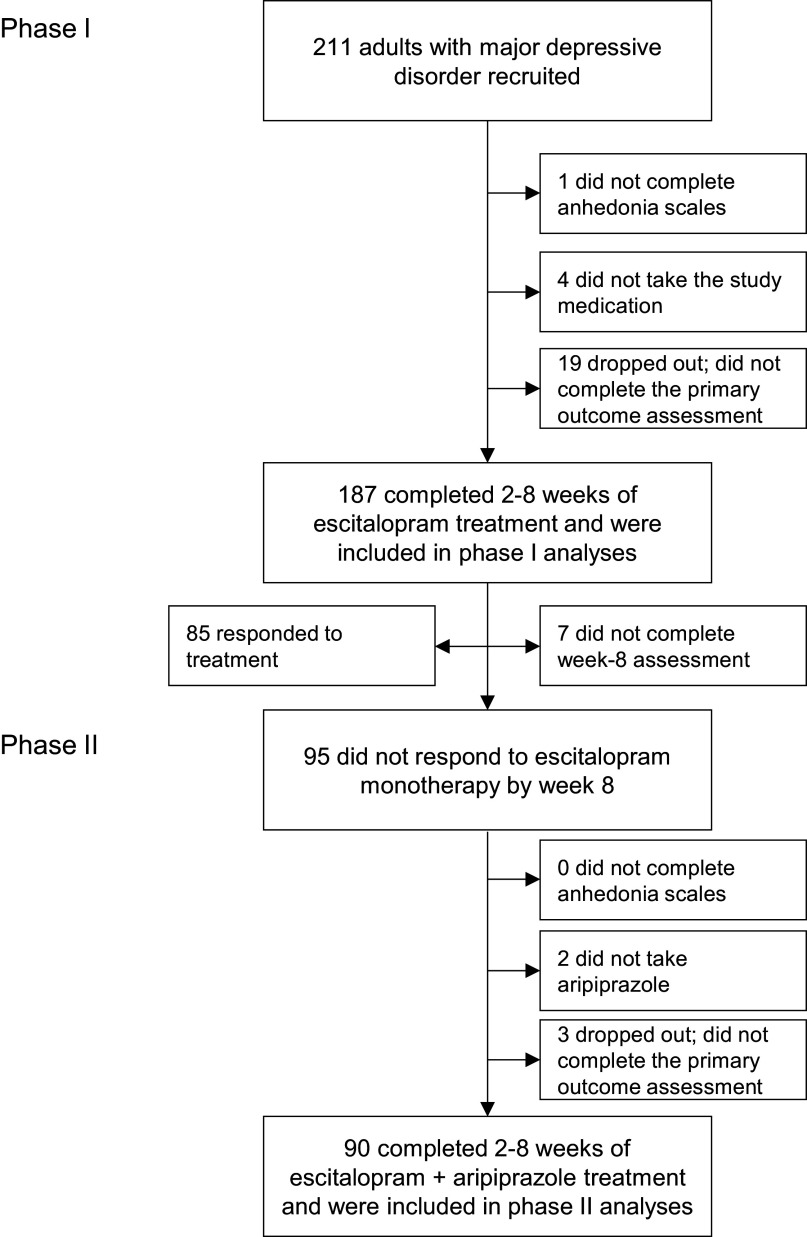
Flow of participants through the study.

Of the 211 participants with MDD recruited to CAN-BIND-1, 187 completed measures of anhedonia and primary outcome measures after 2–8 weeks of treatment with escitalopram (Figure 1; Table 1). Participants who reported more severe anhedonia overall and in the leisure activities domain were less likely to participate in phase I (Table 1). Of the 95 week-8 nonresponders, 90 completed a second set of anhedonia measures and outcome measures after 2–8 weeks of adjunctive treatment with aripiprazole (Figure 1; Table 1). Anhedonia scores were unrelated to participation in phase II. Older participants and those with comorbid anxiety disorders were more likely to participate in the second phase of the clinical trial (Table 2).

**Table 1.** Participant characteristics

	Phase I				Phase II				Comparisons			
	A. Non-participants*n* = 24		B. Escitalopram-treated*n* = 187		C. Non-participants*n* = 5		D. Aripiprazole-treated*n* = 90		A vs. B		C vs. D	
	*n*	%	*n*	%	*n*	%	*n*	%	chi2	*p*	chi2	*p*
Sex												
Female	18	75%	115	61%	5	100%	50	56%				
Male	6	25%	72	39%	0	0%	40	44%	1.66	0.197	3.84	0.050
Race												
Asian	5	21%	27	14%	2	40%	11	12%				
Black	2	8%	9	5%	0	0%	4	4%				
White	14	58%	136	73%	3	60%	70	78%				
Other/mixed	3	13%	15	8%	0	0%	5	6%	2.20	0.532	3.88	0.275
Marital status												
Single/divorced	16	67%	136	73%	5	100%	60	67%				
Married/cohabiting	4	17%	51	27%	0	0%	30	33%	1.24	0.265	2.44	0.119
Employment status												
Unemployed	8	33%	37	20%	0	0%	19	21%				
Employed/student	16	67%	150	80%	5	100%	71	79%	2.33	0.127	1.32	0.251
Comorbid disorders												
Anxiety	12	50%	87	47%	0	0%	42	47%	0.10	0.748	4.18	0.041
Obsessive-compulsive	1	4%	8	4%	0	0%	4	4%	0.00	0.980	0.232	0.630
Substance use	0	0%	8	4%	0	0%	5	6%	1.07	0.302	0.293	0.588
	*mean*	*S.D.*	*mean*	*S.D.*	*mean*	*S.D.*	*mean*	*S.D.*	*t*	*p*	*t*	*p*
Age (years)	35.3	13.5	35.3	12.6	23.8	1.6	36.3	12.7	−0.02	0.983	−2.17	0.032
Depression severity (MADRS)	28.5	5.8	30.1	5.6	31.0	2.8	30.9	5.6	−1.28	0.202	0.04	0.966
Anhedonia	(*n* = 23)											
Hobbies	10.1	3.8	7.7	4.1	6.8	3.0	7.5	4.3	2.71	0.007	−0.35	0.729
Food	10.4	3.2	9.3	4.0	8.8	4.0	8.9	4.1	1.35	0.178	−0.07	0.944
Social	7.9	4.1	6.5	4.0	5.2	3.4	6.4	4.0	1.56	0.121	−0.68	0.500
Sensory	11.2	5.5	9.6	5.6	5.4	6.3	9.9	5.9	1.29	0.197	−1.65	0.103
Total (DARS)	39.7	11.3	33.1	14.4	26.2	15.1	32.7	14.9	2.12	0.036	−0.95	0.344
Total (SHAPS)	31.5	5.5	35.5	5.7	39.8	3.9	36.4	5.6	−2.96	0.004	1.34	0.184

**Table 2.** Anhedonia symptoms and outcomes of treatment with escitalopram monotherapy

Predictor	Effect	95% CI		*p*	q
	*estimate*	*lower*	*upper*		*FDR*
*DARS items*					
Enjoy activities.	0.44	−0.63	1.50	0.424	0.969
Spend time doing activities.	1.24	0.18	2.30	0.022	0.660
Want to do activities.	0.29	−0.77	1.36	0.591	0.969
Interest in activities.	0.82	−0.21	1.86	0.119	0.969
**Activity anhedonia subscale.**	**0.82**	**−0.25**	**1.89**	**0.135**	
Make effort to get food.	−0.10	−1.14	0.93	0.843	0.969
Enjoy food.	0.45	−0.64	1.53	0.419	0.969
Want food.	0.27	−0.78	1.31	0.614	0.969
Want to eat more.	0.86	−0.14	1.86	0.091	0.969
**Food anhedonia subscale.**	**0.49**	**−0.56**	**1.53**	**0.362**	
Socializing makes me happy.	0.19	−0.89	1.26	0.734	0.969
Interested in socializing.	−0.28	−1.37	0.81	0.619	0.969
Plans/initiates social activities.	−0.08	−1.12	0.96	0.885	0.969
Actively participates in social activities.	0.09	−0.95	1.14	0.860	0.969
**Social anhedonia subscale**	**−0.02**	**−1.10**	**1.06**	**0.973**	
Seek sensory experiences.	1.21	0.17	2.26	0.023	0.667
Excited thinking of sensory experiences.	1.10	0.06	2.14	0.038	0.969
Savor sensory experiences.	0.82	−0.25	1.89	0.133	0.969
Want sensory experiences.	0.87	−0.17	1.90	0.100	0.969
Make effort to get sensory experiences.	0.94	−0.12	2.00	0.083	0.969
**Sensory anhedonia subscale**	**1.14**	**0.08**	**2.20**	**0.034**	
**DARS total score**	**0.84**	**−0.24**	**1.91**	**0.127**	
*SHAPS items*					
Enjoy a TV/radio program.	0.79	−0.34	1.91	0.172	0.969
Enjoy being with family/friends.	0.58	−0.57	1.74	0.321	0.969
Pleasure in hobbies.	0.12	−0.99	1.23	0.831	0.969
Enjoy favorite meal.	0.25	−0.94	1.45	0.679	0.969
Enjoy bath/shower.	1.25	0.18	2.32	0.022	0.660
Pleasure in scent/smell.	1.05	−0.03	2.12	0.056	0.969
Enjoy smiling faces.	0.87	−0.19	1.93	0.107	0.969
Enjoy appearance.	0.38	−0.72	1.47	0.500	0.969
Enjoyed reading.	0.43	−0.70	1.55	0.457	0.969
Enjoy a drink.	0.71	−0.40	1.83	0.210	0.969
Pleasure in small things.	0.02	−1.12	1.16	0.969	0.969
Enjoy beautiful landscape.	1.08	−0.02	2.17	0.053	0.969
Pleasure in helping others.	0.57	−0.51	1.65	0.298	0.969
Pleasure in praise.	0.27	−0.80	1.34	0.617	0.969
**SHAPS sensory subscale**	**1.50**	**0.43**	**2.57**	**0.006**	
**SHAPS total score**	**1.30**	**0.13**	**2.47**	**0.030**	

*Note:* The effects of baseline predictors on outcome are presented in units of MADRS score per one standard deviation of each predictor.

Abbreviations: 95%CI = 95% confidence interval of the effect estimate; *p*, two-sided *p*-value; q, false discover rate (FDR) q-value; DARS, Dimensional Anhedonia Rating Scale; SHAPS, Snaith-Hamilton Pleasure scale.

Bolded entries represent subscales and total scores.

### Prediction of response to escitalopram

Of the four DARS subscales, sensory anhedonia predicted a worse treatment outcome with escitalopram with nominal statistical significance (Table 2). No subscale predicted significantly after correcting for multiple testing. Six of the 31 DARS and SHAPS items predicted a worse treatment outcome with escitalopram with nominal significance; five of these items indexed sensory experiences (Table 2). No item-level prediction was significant after correction for multiple comparisons. A subscale constructed of the three sensory items of SHAPS predicted escitalopram treatment outcome more strongly than its DARS counterpart (*b* = 1.50, 95%CI 0.43 to 2.57, *p* = 0.006). The DARS total anhedonia score was not significantly related to escitalopram monotherapy outcomes. The SHAPS total anhedonia score predicted worse outcomes of treatment with escitalopram with an effect size smaller than that of its sensory subscale (Table 2).

### Prediction of response to adjunctive aripiprazole

Of the four DARS subscales, food and social anhedonia dimensions predicted outcomes of treatment with adjunctive aripiprazole (Table 3). In both cases, higher levels of anhedonia prior to adding aripiprazole were associated with worse outcomes of the adjunctive treatment. Both predictions remained significant after correcting for multiple testing. Ten of the 31 DARS and SHAPS items predicted a worse outcome of treatment with adjunctive aripiprazole at nominal significance, and 6 of them were significant after FDR correction for multiple testing; all six FDR-significant items concerned food or social anhedonia (Table 3). The total anhedonia scores of both DARS and SHAPS at week 8 predicted less improvement with adjunctive aripiprazole, albeit with smaller effect sizes than the scores in the food and social domains (Table 3).

**Table 3.** Anhedonia symptoms and outcomes of treatment with adjunctive aripiprazole

Predictor	Effect	95%CI		*p*	q
	*Estimate*	*Lower*	*Upper*		*FDR*
*DARS items*					
Enjoy activities.	0.58	−0.87	2.03	0.437	0.958
Spend time doing activities.	0.04	−1.34	1.41	0.958	0.958
Want to do activities.	1.08	−0.23	2.39	0.106	0.958
Interest in activities.	0.75	−0.57	2.08	0.266	0.958
**Activity anhedonia subscale.**	**0.75**	**−0.67**	**2.18**	**0.301**	
Make effort to get food.	1.24	−0.11	2.60	0.072	0.958
Enjoy food.	2.28	1.08	3.48	0.000	0.000
Want food.	1.93	0.72	3.15	0.002	0.050
Want to eat more.	2.17	0.99	3.35	0.000	0.000
**Food anhedonia subscale.**	**2.52**	**1.25**	**3.80**	**0.000**	
Socializing makes me happy.	2.09	0.85	3.33	0.001	0.027
Interested in socializing.	2.49	1.18	3.80	0.000	0.000
Plans/initiates social activities.	1.44	−0.04	2.93	0.057	0.958
Actively participates in social activities.	2.15	0.75	3.54	0.003	0.072
**Social anhedonia subscale**	**2.56**	**1.16**	**3.96**	**0.000**	
Seek sensory experiences.	−0.24	−1.65	1.16	0.732	0.958
Excited thinking of sensory experiences.	0.54	−0.80	1.89	0.428	0.958
Savor sensory experiences.	0.68	−0.61	1.97	0.302	0.958
Want sensory experiences.	1.33	0.10	2.56	0.035	0.700
Make effort to get sensory experiences.	−0.28	−1.65	1.09	0.691	0.958
**Sensory anhedonia subscale.**	**0.50**	**−0.87**	**1.87**	**0.476**	
**DARS total score**	**1.94**	**0.47**	**3.41**	**0.010**	
*SHAPS items*					
Enjoy a TV/radio program.	0.64	−0.75	2.04	0.367	0.958
Enjoy being with family/friends.	1.83	0.65	3.00	0.002	0.050
Pleasure in hobbies.	−1.29	−2.65	0.07	0.062	0.958
Enjoy favorite meal.	2.42	1.10	3.74	0.000	0.000
Enjoy bath/shower.	0.57	−0.90	2.05	0.446	0.958
Pleasure in scent/smell.	0.97	−0.43	2.37	0.176	0.958
Enjoy smiling faces.	0.90	−0.44	2.23	0.187	0.958
Enjoy appearance.	1.56	0.20	2.92	0.025	0.550
Enjoyed reading.	1.52	0.14	2.90	0.031	0.651
Enjoy a drink	0.85	−0.66	2.36	0.270	0.958
Pleasure in small things.	0.55	−0.86	1.96	0.442	0.958
Enjoy beautiful landscape.	1.81	0.40	3.21	0.012	0.276
Pleasure in helping others.	0.19	−0.98	1.36	0.749	0.958
Pleasure in praise.	0.90	−0.31	2.10	0.145	0.958
**SHAPS sensory subscale**	**1.90**	**0.29**	**3.52**	**0.020**	
**SHAPS total score**	**2.45**	**0.80**	**4.10**	**0.004**	

*Note:* The effects of week-8 predictors on outcome are presented in units of MADRS score per one standard deviation of each predictor.

Abbreviations: 95%CI = 95% confidence interval of the effect estimate; *p*, two-sided *p*-value; DARS, Dimensional Anhedonia Rating Scale; SHAPS, Snaith-Hamilton Pleasure scale.

Bolded entries represent subscales and total scores.

### Complementarity of anhedonia and interest–activity symptom dimensions

**Figure 2.**
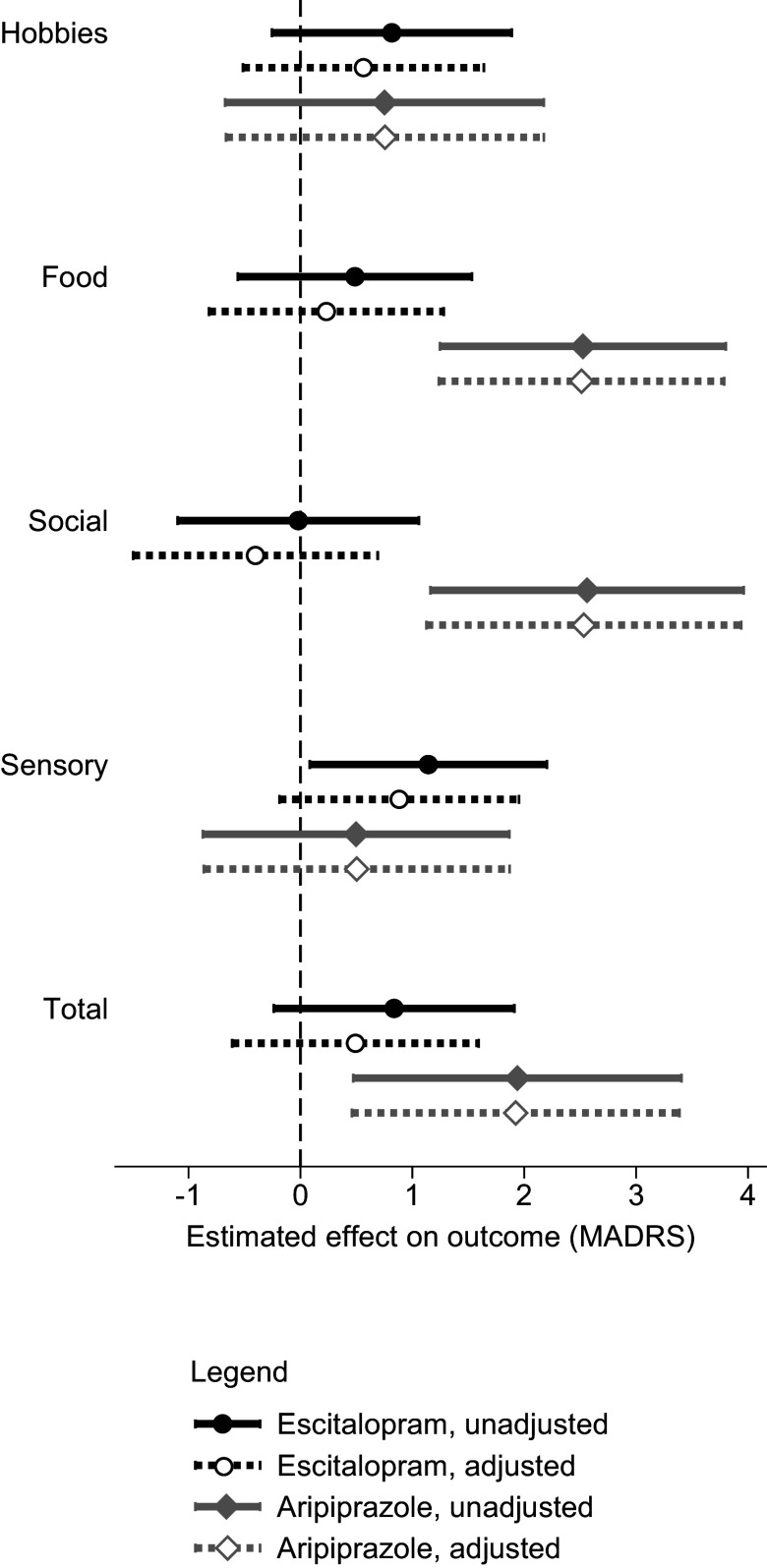
The effects of anhedonia and interest–activity dimensions on treatment outcomes. The symbols and horizontal lines represent the estimates and 95% confidence intervals of the Montgomery-Åsberg Depression Rating Scale (MADRS) score points difference in outcomes per one standard deviation of the predictor.

While the total DARS and SHAPS scores were moderately correlated at baseline (Pearson product–moment *r* = 0.52) and strongly at week 8 (*r* = 0.67), their correlations with the interest–activity symptom dimension were weaker at baseline (*r* = 0.39–0.43) and at week 8 (*r* = 0.39–0.48; Table 4). When baseline anhedonia and interest–activity dimensions were entered into the same mixed-effects model predicting the outcome of treatment with escitalopram, the effect of sensory anhedonia was attenuated (Figure 2) but the effect of interest–activity remained significant (beta 1.49, 96%CI 0.17 to 2.83, *p* = 0.027). While the DARS sensory anhedonia subscale became nonsignificant with the inclusion of the interest–activity dimension, the SHAPS sensory anhedonia subscale complemented the interest–activity symptom dimension to improve treatment outcome prediction with a significant unique effect (beta 1.26, 95%CI 0.17 to 2.34, *p* = 0.023). A combined predictor obtained by averaging the standardized DARS–SHAPS sensory anhedonia and the interest–activity dimension scores predicted escitalopram treatment outcomes with the largest effect size of all predictors examined (beta = 2.39, 95%CI 1.18 to 3.59, *p* < 0.001).

**Table 4.** Correlations between anhedonia and depressive symptoms measures at baseline and at week 8

	*Anhedonia*	Food	Social	Sensory	Total (DARS)	Total (SHAPS)	Depressive symptoms	
	Hobbies						Interest-activity	Total severity
**A. Baseline (*n* = 187)**								
*Anhedonia*								
Hobbies (DARS)	1.00							
Food (DARS)	0.56	1.00						
Social (DARS)	0.53	0.49	1.00					
Sensory (DARS)	0.52	0.52	0.61	1.00				
Total (DARS)	0.79	0.78	0.80	0.85	1.00			
Total (SHAPS)	0.42	0.47	0.47	0.36	0.52	1.00		
*Depressive symptoms*								
Interest-activity (MADRS, QIDS-SR)	0.33	0.34	0.39	0.35	0.43	0.39	1.00	
Total severity (MADRS)	0.26	0.30	0.31	0.28	0.35	0.38	0.65	1.00
**B. Week 8 (*n* = 90)**								
*Anhedonia*								
Hobbies	1.00							
Food	0.42	1.00						
Social	0.54	0.44	1.00					
Sensory	0.65	0.52	0.63	1.00				
Total (DARS)	0.81	0.72	0.79	0.90	1.00			
Total (SHAPS)	0.52	0.59	0.47	0.59	0.67	1.00		
*Depressive symptoms*								
Interest-activity (MADRS, QIDS-SR)	0.33	0.27	0.29	0.35	0.39	0.48	1.00	
Total severity (MADRS)	0.42	0.39	0.32	0.44	0.49	0.54	0.74	1.00

*Note:* Numbers in the table are the Pearson’s product–moment correlation coefficients. Panel A shows correlations at baseline, based on 187 participants; Panel B shows correlations at week 8, based on 90 participants.

Abbreviations: DARS, Dimensional Anhedonia Rating Scale; SHAPS, Snaith–Hamilton Pleasure Scale; MADRS, Montgomery-Åsberg Depression Rating Scale; QIDS-SR, Quick Inventory for Depressive Symptomatology, Self-Report.

When week-8 anhedonia and interest–activity dimensions were entered into a model predicting outcomes of treatment with adjunctive aripiprazole, the effect of food and social anhedonia remained unchanged (Figure 2). The interest–activity symptom dimension was not significantly related to the outcome of treatment with adjunctive aripiprazole.

## Discussion

In a clinical trial of a serotonin-reuptake inhibitor antidepressant followed by adjunctive treatment with a partial dopamine agonist in MDD, reward domain-specific dimensions of anhedonia were more potent and specific predictors of treatment outcomes than total anhedonia scores. Sensory anhedonia was associated with poor response to serotonergic antidepressant monotherapy. Food and social anhedonia were associated with poor response to adjunctive dopaminergic treatment. Interest–activity symptom dimension and anhedonia-specific measures played complementary roles in predicting outcomes of antidepressant monotherapy and adjunctive treatment.

The finding that the sensory dimension of anhedonia predicts the outcome of treatment with a serotonin-reuptake inhibitor antidepressant is new. We are not aware of any prior study testing the effect of specific dimensions of anhedonia on response to antidepressant treatment. Several previous studies reported that anhedonia predicted worse treatment outcome in people with MDD, but none of these studies used a specific measure of anhedonia or monotherapy with a serotonin-reuptake inhibitor antidepressant. Instead, they used factor scores including depressive symptoms and other constructs in adult inpatients or adolescents selected for nonresponse to previous antidepressants and receiving multimodal treatments (McMakin et al., 2012; Vrieze et al., 2014). In one study, the predictor labeled as ‘anhedonia’ included early separation from parents (Vrieze et al., 2014). Another study used a factor derived from items of a depression rating scale (McMakin et al., 2012). The only previous publication reporting a relationship between a specific anhedonia measure and outcome of antidepressant monotherapy in an unselected sample of patients with MDD was a previous analysis of a subset of the CAN-BIND-1 sample, finding that one of two anhedonia measures was associated with treatment outcome (Dunlop et al., 2020). In this context, the notion that anhedonia is associated with worse outcomes of antidepressant treatment needs to be revised. Reward-domain-specific effects provide a new framework that may help reconcile previously inconsistent findings.

Among people who did not respond to a monotherapy with a serotonin-reuptake inhibitor antidepressant, food and social dimensions of anhedonia predicted nonresponse to adjunctive partial dopamine agonist aripiprazole. To our best knowledge, this finding is novel as no previously published study tested dimensions of anhedonia as predictors of the outcome of adjunctive treatment with a partial dopamine agonist. The finding may appear surprising in the context of a previously reported good response to adjunctive dopamine-enhancing medications among people with marked anhedonia (Dormegny-Jeanjean et al., 2023). However, the present finding concords with studies that used a behavioral assessment of hedonic function with the probabilistic reward task and found that better hedonic function was associated with better responses to bupropion, a dopamine and norepinephrine reuptake inhibitor (Ang et al., 2020), and to pramipexole, a D3-prefenrential dopamine agonist (Whitton et al., 2020). These studies differed from the present work both in the measure of anhedonia and in the choice of dopaminergic medication. The specificity of prediction to the food and social domains of anhedonia may reflect the distinct neurocircuitry and pharmacology of nutritive and affiliative reward systems, with greater involvement of the insula, the septo-hypothalamic circuits, and oxytocine (Bortolini et al., 2021; Snowdon-Farrell et al., 2025; Uher et al., 2006). It is possible that the preserved function of these nondopaminergic mechanisms is a prerequisite for the efficacy of dopaminergic therapeutics. However, in the absence of direct comparability among published studies, future work is needed to map the relevance of reward domains and anhedonia dimensions to the therapeutic effects of dopamine-enhancing treatments.

Modern research on anhedonia has emphasized the reward process, distinguishing between reward anticipation, enjoyment, and learning (Berridge & Robinson, 2003; Pizzagalli, 2022). While the DARS items index the various reward processes, the pattern of item-level results in the present analyses clearly shows that the prediction of treatment response is specific to the domain of reward rather than the reward process. This corresponds to the results of factor analyses of anhedonia scales in clinical and nonclinical human samples (Arrua-Duarte et al., 2019; Case et al., 2022; Fresán & Berlanga, 2013; Rizvi et al., 2015; Zhang et al., 2021). While the mapping of all stages of the reward process has clear advantages in the examination of the underlying neural and pharmacological mechanisms, we suggest that additional attention to the domain of reward may be needed to tap clinically relevant relationships.

Activities anhedonia predicted participation in the treatment trial. We are not aware of any published studies examining the relationship between anhedonia dimensions and research participation or treatment uptake. The fact that participants with more profound loss of interest and pleasure in leisure activities were more likely to participate in a treatment study may reflect the aversive nature of activity anhedonia, which incites intrinsic motivation for treatment.

The centrality of anhedonia to the depressive experience leads to aspects of anhedonia featuring in numerous depression-related concepts. One of them is the interest–activity symptom dimensions, which emerged from factor analysis of depression rating scales and proved to be a potent and reproducible predictor of antidepressant treatment outcomes (Uher et al., 2012; Uher et al., 2020). While some authors reference interest–activity symptoms as equivalent to anhedonia, the inclusion of concentration and activity levels in addition to interest distinguishes interest–activity from anhedonia measures. The present analysis suggests that interest–activity symptom dimension and anhedonia-specific measures play distinct and complementary roles in predicting depression treatment outcomes. The interest–activity symptoms are the strongest predictor of the outcome of monotherapy with a serotonin-reuptake inhibitor, but, unlike anhedonia dimensions, they are unrelated to the outcomes of adjunctive treatment with a partial dopamine agonist. While both interest–activity and sensory anhedonia predict response to serotonin-reuptake inhibiting antidepressant, their contributions to the prediction are complementary rather than overlapping. As a result, the combination of interest–activity symptoms and sensory anhedonia can be leveraged as a potent predictor of response to selective serotonin reuptake inhibitors.

Through the inclusion of two measures of anhedonia, SHAPS and DARS, the present investigation can inform measure selection for future research. As a full scale, SHAPS provided a stronger prediction of treatment outcomes. The SHAPS total score and its three-item sensory anhedonia subscale predicted outcomes in both stages of the clinical trial. However, DARS provides better coverage of reward domains and processes. The food and social anhedonia subscales of DARS were the strongest predictors of adjunctive treatment with a partial dopamine agonist. With these complementary advantages, the preference for one or the other measure will depend on the primary goal of a particular project. In studies where anhedonia is a primary focus, the availability of both measures will strengthen the coverage of this multifaceted concept.

The results of this study have to be interpreted in the context of significant limitations. They are based on secondary analyses in a medium-sized sample with limited statistical power. The predictive effect of the DARS sensory anhedonia dimension was not statistically significant after correction for multiple testing. The SHAPS sensory anhedonia subscale, which uniquely predicted the outcomes in both treatment stages, has not been previously validated. While the results are consistent across the two anhedonia rating scales, this is not a replication as both scales were completed by the same individuals. Therefore, an independent replication is warranted.

In conclusion, reward domain-specific dimensions of anhedonia improve the prediction of treatment outcomes. The inability to enjoy sensory experiences may be distinct from the broader concept of anhedonia in its role as a predictor of antidepressant treatment outcomes. Loss of interest and pleasure in food and social activities is relevant to the success of adjunctive dopaminergic treatment. Mapping the patterns of relationships between aspects of anhedonia, interest–activity and treatment outcomes will help improve multivariate prediction and personalized treatment selection.
